# Exploring the potential of tomato juice (*Solanum lycopersicum* L.) patch for tooth bleaching

**DOI:** 10.34172/joddd.41042

**Published:** 2024-09-07

**Authors:** Laifa Annisa Hendarmin, Berliana Novianita, Yuni Anggraeni, Khairunissa Febriyanti

**Affiliations:** ^1^Medical Biology Department, Faculty of Medicine, Universitas Islam Negeri Syarif Hidayatullah Jakarta, Banten, Indonesia; ^2^Molecular Diagnostics and Research Center, Faculty of Medicine, Universitas Islam Negeri Syarif Hidayatullah Jakarta, Banten, Indonesia; ^3^Graduate School of Health and Drug Sciences, Université Paris-Saclay, Orsay, France; ^4^Faculty of Health Sciences, Universitas Islam Negeri Syarif Hidayatullah Jakarta, Banten, Indonesia

**Keywords:** Dental bleaching, Dental patch, *Solanum lycopersicum* L, Tomato

## Abstract

**Background.:**

Tomato, renowned for its tooth-whitening properties due to its hydrogen peroxide and peroxidase enzyme content, requires formulation for enhanced applicability. This study assessed the dental whitening efficacy of a patch containing tomato juice as the active ingredient.

**Methods.:**

Tomato juice patches were formulated at concentrations of 55%, 60%, and 65%. A control group (C) containing a 12% hydrogen peroxide patch was also included. A matrix layer of the patches was prepared using the solvent casting method at 40 for 18 h, with Tegaderm^TM^ applied as the backing layer. For the dental whitening procedure, each group of patches was applied to the labial surface of anterior stained teeth (n=6/group) for 3 h, repeated 14 times. Tooth brightness was evaluated qualitatively and quantitatively using digital dental photo CIEL*a*b* analysis. Meanwhile, enamel surfaces were examined under a scanning electron microscope (SEM). Repeated-measures analysis of variance (ANOVA) was employed for statistical analysis.

**Results.:**

The application of tomato juice patches led to enhanced tooth brightness. The patch containing 65% tomato juice significantly improved stained tooth brightness (*P*<0.05). SEM analysis revealed less enamel erosion with the 65% tomato juice patch compared to the 12% hydrogen peroxide patch.

**Conclusion.:**

Tomato juice patches effectively increased tooth brightness while minimizing demineralization. Further research is needed to optimize formulation and explore its potential.

## Introduction

 Tooth staining can have multifactorial causes, including chromogens derived from foods and habits directly related to oral activity, such as smoking, chewing, and drinking coffee and tea. These chromogens penetrate the tooth pellicle, resulting in discoloration that mirrors the chromogen. Polyphenols in food and beverages are suspected to be the primary culprit behind tooth staining.^1^

 According to Tin-Oo et al.,^2^ 52.8% of the patients in Hospital Universiti Sains Malaysia reported dissatisfaction with their dental appearance, particularly concerning dental color. Consequently, tooth-whitening procedures are the most sought-after treatment by patients (48%).

 Tooth-whitening products have evolved into various dosage forms over time. The active ingredient in many of these products is hydrogen peroxide (H_2_O_2_), delivered either as hydrogen or carbamide peroxide. However, these products can lead to side effects such as gingival irritation and tooth sensitivity. Consequently, experts have explored alternatives to these conventional dental bleaching materials, seeking safer and more cost-effective options. Tomatoes emerge as a promising natural whitening agent as they contain peroxide compounds.^3^

 Tomatoes contain hydrogen peroxide and peroxidase enzymes. Hydrogen peroxide penetrates through the enamel to the dentinal tubules, acting as a potent oxidizing agent that generates highly reactive free radicals. These compounds effectively damage dye molecules, neutralizing their color and producing a bleaching effect. Peroxidase accelerates the action of hydrogen peroxide in reducing color.^4^ Lumuhu et al^5^ compared the effectiveness of tomato juice and apple juice as natural tooth-whitening agents by immersing teeth in pure juice. The findings indicated that tomato and apple juices demonstrated whitening effects, with tomato juice proving more effective than apple juice. The promising potential of tomatoes as a natural tooth whitener has led researchers to explore their formulation in tooth-whitening patches. This study aimed to develop a tooth-whitening patch incorporating tomato juice as the active ingredient. Additionally, it sought to assess its whitening and demineralizing effects.

## Methods

###  Patch preparation

Table 1 shows the patch ingredients. All materials were weighed, and hydroxypropyl methylcellulose (HPMC) was dissolved in a portion of distilled water (M1). Polyvinylpyrrolidone (PVP) was also dissolved in another portion of distilled water until fully dissolved (M2). M2 was then combined with M1 while stirring until it became homogeneous, and glycerine was added. Finally, in formulas F1 (55% tomato juice), F2 (60% tomato juice), and F3 (65% tomato juice), the tomato juice was incorporated into the solution. For the control group formula (C), hydrogen peroxide was added and stirred until it became homogeneous. The resulting film-forming solution was then poured into a calibrated mold and dried at 40°C for 18 h to form films. These films were then separated from the mold and placed in an airtight container containing silica until a constant weight was achieved. The films were subsequently measured according to size. Some of the films were coated with a Tegaderm^TM^ backing membrane. The functional properties of the patches, including surface pH, folding endurance, and residence time, were evaluated in a previous study.^6^

**Table 1:** Patch formulas

**Ingredients**	**Patch Formula (gr)**			
	**F1**	**F2**	**F3**	**C**
100% Tomato juice	16.5	18.0	19.5	-
30% Hydrogen peroxide	-	-	-	1.8
HPMC	2.1	2.1	2.1	2.1
PVP	0.9	0.9	0.9	0.9
Glycerin	1.5	1.5	1.5	1.5
Aquadest ad.	30	30	30	30

###  Tooth preparation 

 In this study, 30 extracted teeth were sourced from patients undergoing orthodontic treatment, adhering to specific criteria ensuring the tooth’s integrity. Each tooth was cleaned, and a layer of clear white nail polish was applied to every root to prevent the tea solution from penetrating the dentinal tubule during the staining process. Subsequently, each tooth was immersed in a tea solution for 12 days, with daily replacement of the tea solution. After each treatment cycle, the tooth’s color was evaluated both qualitatively using a shade guide and quantitatively utilizing the digital dental photo technique of CIEL*a*b* analysis.^5^ The teeth were then divided into five groups (untreated, F1, F2, F3, and control group), each comprising six teeth.

###  Dental whitening procedure

 Artificial saliva was prepared using the Afnor method to replicate the conditions of the human mouth, incorporating the ingredients presented in Table 2. The four patch formulas were then applied to four distinct groups of teeth. Each tooth was positioned upright with its roots embedded in plasticine. Each tooth was moistened with 50 μL of artificial saliva, and the patch was affixed with slight pressure until securely in place. The application lasted three hours, with humidity maintained by periodically dripping 3 mL of artificial saliva every 10 minutes. Subsequently, the teeth were rinsed under running water, brushed, and dried at room temperature. This procedure was repeated 14 times. It is typically recommended that commercial tooth-whitening strips should be worn twice a day for 30 minutes over 14 days.^7^ However, in this experiment, we optimized the patch application duration to three hours per day. This decision was based on our patch formulation’s in vitro residence time of up to three hours on teeth before gradual dissolution commenced.^6^

**Table 2:** Artificial saliva made by the Afnor method

**Ingredients**	**g/L**
Na_2_HPO_4_	0.26
KSCN	0.33
NaCl	6.00
KH_2_PO_4_	0.20
KCl	1.20
NaHCO_3_	1.50
HCl	Adjust pH 6.8
Distilled water	added to 1 litre

###  Quantitative analysis of dental brightness

 In this study, tooth brightness was measured quantitatively using the CIEL*a*b* measurement system, which defines color in three dimensions. Firstly, luminosity or value indicates the luminosity relative to the color and encompasses the quantity of grey. Secondly, the shade reflects the object’s true color, such as green, red, blue, or yellow. Finally, chroma is the concentration or intensity of the shade. This method uses three parameters: L*, a*, and b*. The color location within this system is determined by the coordinates of L*, a*, and b*. The L* parameter ranges from 0 to 100 (black to white), revealing the reflective light that produces achromatic colors ranging from white to grey to black. The a* parameter ranges from 0 to + 80 (red) and 0 to -80 (green), indicating the mixed chromatic color of red-green. Similarly, the b* parameter ranges from 0 to + 70 (yellow) and 0 to -70 (blue), representing the mixed chromatic color blue-yellow.^8,9^

 The digital dental photo technique of CIEL*a*b* analysis was employed in this study. Before and after the treatment, samples were photographed using a DSLR camera under consistent place, position, and lighting conditions for each shoot. The resulting photos were then analyzed using Adobe Photoshop software with L*a*b* color mode. This method is effective and efficient for assessing changes in enamel color on teeth. From the analysis of the results, values for L*, a*, and b* were obtained and used in a formula to calculate the final score. A higher score indicates a brighter tooth color. The formula used is ∆E*ab = [(∆L*)^2^ + (∆a*)^2^ + ∆(b*)^2^]^1/2^, where ΔE*ab represents the total color change; ΔL is the change in lightness, Δa is the change in chroma red-green, and Δb is the change in chroma blue-yellow.^5^

###  Qualitative analysis of dental brightness

 This analysis was conducted before and after treatment by three independent observers using a shade guide comprising 16 tooth colors to assess tooth brightness qualitatively. The shade guide is a color scale and consists of artificial teeth with various color codes. A1-A4 represents colors in the red-to-brown spectrum; B1-B4 indicates colors in the red-to-yellow spectrum; C1-C4 denotes colors in the grey spectrum; and D2-D4 represents colors from red to ash.Before measurement, the shade guide’s colors were arranged from the brightest to the darkest, as detailed in Table 3. A higher score indicates that the teeth appeared darker.^10^

**Table 3:** Shade guide scores

**Code**	**Score**
B1	1
A1	2
B2	3
D2	4
A2	5
D3	6
B3	7
A3.5	8
B4	9
C3	10
A4	11
C4	12

###  Tooth demineralization evaluation

 After rinsing the teeth under running water to ensure cleanliness, they were gold-coated and examined for morphology using an SEM, following established procedures.^11,12^

###  Statistical analysis

 Thedata were analyzed using SPSS 22 (ID: 213021). Descriptive statistics, including means and standard deviations, were calculated for each group. Repeated-measures ANOVA was employed to assess differences in tooth brightness. In this study, a significance level of P ≤ 0.05 was considered.

## Results

###  Tomato juice patches 

 Visually, all tomato juice patches (F1, F2, F3) exhibited similar physical characteristics, appearing as reddish-brownish white and opaque patches. The upper surface texture was rough, while the lower surface was smooth and flat.

**Figure 1:**
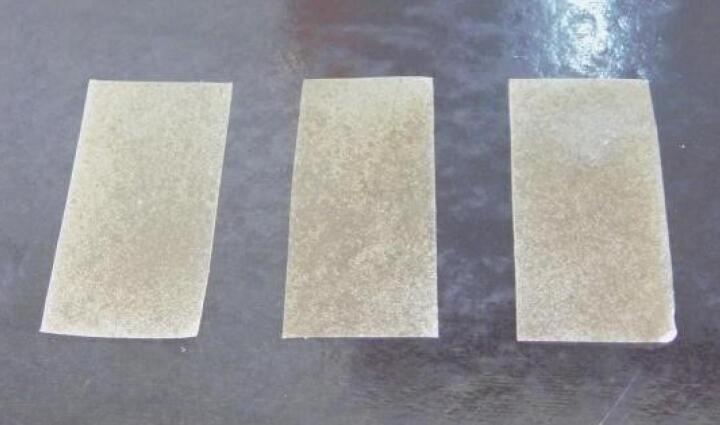
Macroscopic features of the tomato juice patches (F1 = 55%; F2 = 60%; F3 = 65%)

 As shown in Figure 1, the intensity of the brown color is due to the color of the tomato juice. The varying concentrations of tomato juice in the three formulas did not affect the intensity of the film color. Additionally, the films were thin, supple, and not brittle, and emitted the distinctive aroma of tomatoes.

###  Preparation of the dental specimens 

 The teeth were stained to make them darker to facilitate the observation of their brightness enhancement by applying the tomato juice patch. Black tea was selected as the staining agent in this study since, empirically, individuals are aware that regular tea consumption can lead to tooth discoloration. This choice aligns with prior research indicating that the staining effect of tea surpasses that of other beverages such as cola and soy sauce.^13^

**Figure 2:**
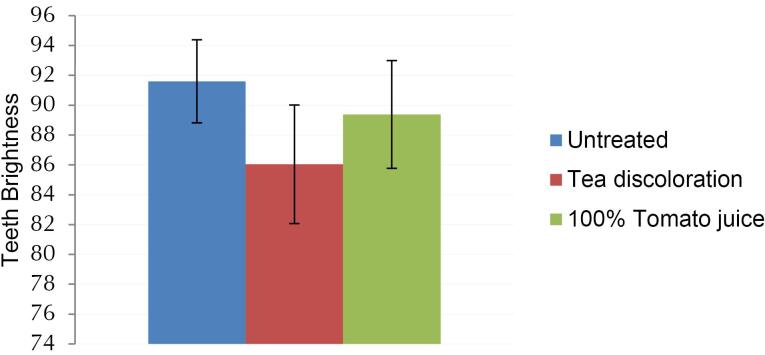
Dental bleaching property of tomato juice (*P* < 0.01)

 The tooth staining process involved immersing them in a tea solution for 12 days. The teeth were then divided into five test groups, each comprising six teeth. The results of this staining process on 30 teeth revealed a significant darkening of tooth color quantitatively (*P* < 0.01), as illustrated in Figure 2.

###  Dental brightness evaluation

 Furthermore, before assessing the bleaching potential of the tomato juice patch, an observational study was conducted to determine the tooth-whitening effect of pure tomato juice. This involved soaking stained teeth in pure tomato juice for 42 hours, matching the observation time of the tomato juice patch applied to the teeth 14 times, where each application lasted 3 hours.

 Based on the tooth brightness evaluation scores shown in Figure 2, it is evident that pure tomato juice exhibited a dental bleaching effect. The test was conducted using a quantitative method employing digital dental photo CIEL*a*b analysis. The results indicated a significant increase in the brightness of the stained teeth (*P* < 0.01).

**Figure 3:**
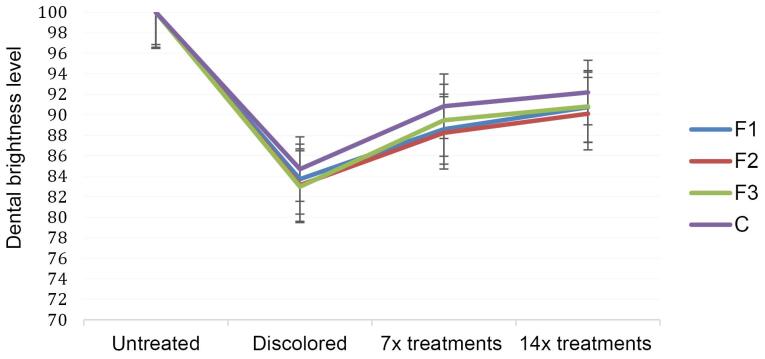
CIEL*a*b analysis of dental brightness after treatment with various concentrations of tomato juice patch (F1 = 55%; F2 = 60%; F3 = 65% tomato juice patch; C = 12% hydrogen peroxide; *P* < 0.05)

 Following the tooth discoloration process, patches F1 (55% tomato), F2 (60% tomato), F3 (65% tomato), and C (hydrogen peroxide) were applied 14 times in separate test groups. Dental bleaching evaluation was performed both qualitatively and quantitatively. In quantitative analysis, as shown in Figure 3, it was observed that all patch formulas significantly enhanced dental brightness (*P* < 0.05) after 7 and 14 applications. The application of the 65% tomato juice patch 14 times demonstrated the most significant increase in dental brightness and was the closest match to the brightness achieved with the 12% hydrogen peroxide application.

**Figure 4:**
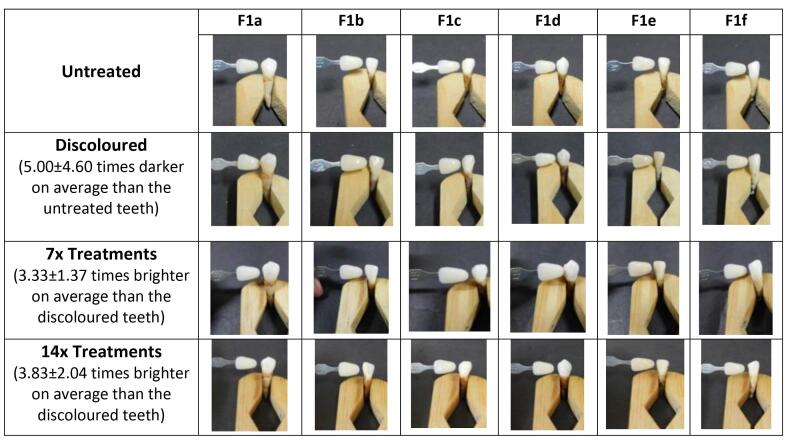
Qualitative analysis of dental brightness after treatment with 55% tomato juice patch (F1)

**Figure 5:**
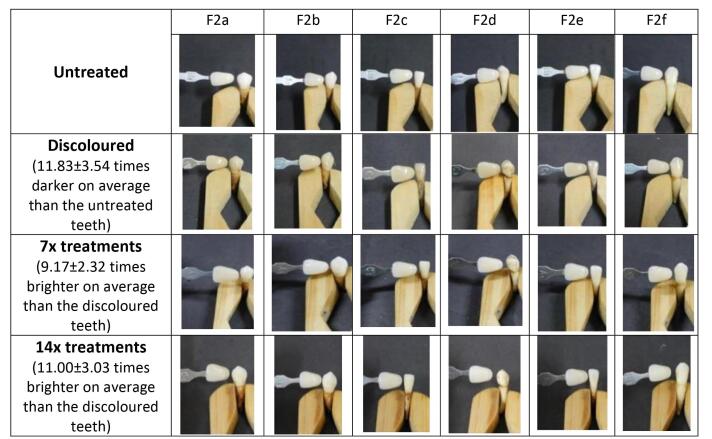
Qualitative analysis of dental brightness after treatment with 60% tomato juice patch (F2)

**Figure 6:**
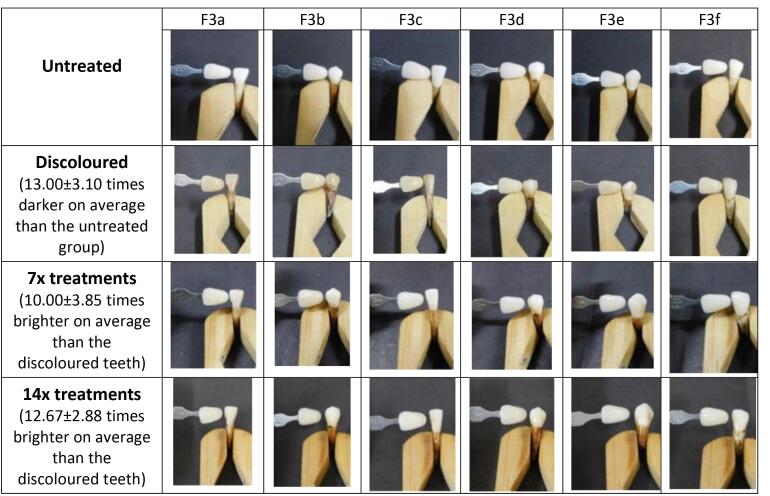
Qualitative analysis of dental brightness after treatment with 65% tomato juice patch (F3)

**Figure 7:**
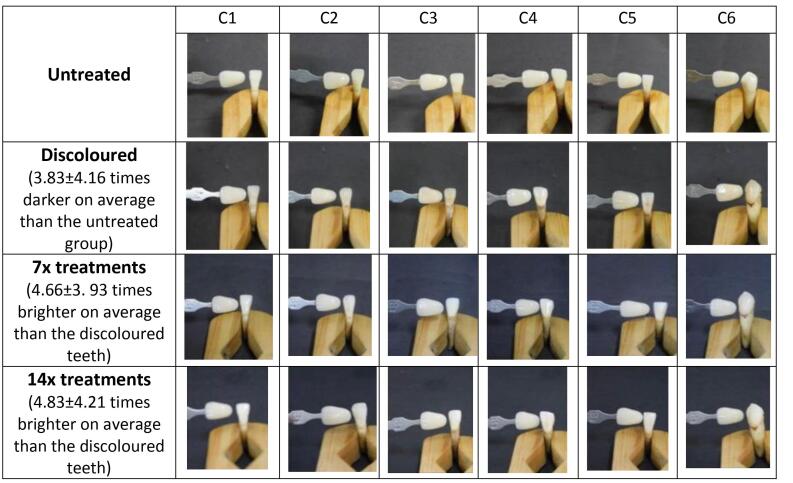
Qualitative analysis of dental brightness after treatment with 12% hydrogen peroxide patch (C)

 In qualitative analysis, scores were assigned to each tooth via three independent observations (Table 3). The scores for discolored teeth and those treated with the patch for 14 applications were calculated to determine each treatment’s mean value and SD. It was observed that the F1 patch increased tooth brightness by a factor of 3.83, as shown in Figure 4. This improvement was lower compared to the F2 and F3 patches, which enhanced tooth brightness by 11 and 12.67 times, respectively (Figures 5 and 6). In comparison, the 12% hydrogen peroxide patch resulted in teeth becoming 4.83 times whiter (Figure 7).

###  Dental demineralization analysis

**Figure 8:**
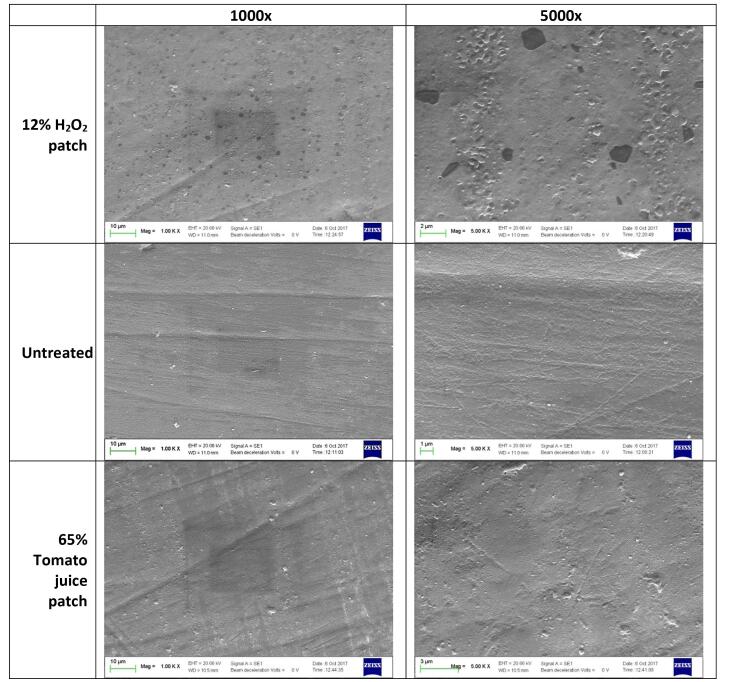
Demineralization evaluation using scanning electron microscopy (SEM)

 Tooth demineralization after patch application was analyzed by examining enamel morphology using a scanning electron microscope (SEM). It was observed that the enamel treated with the 12% hydrogen peroxide patch exhibited greater porosity compared to the enamel treated with the 65% tomato juice patch (Figure 8), indicating that the application of the tomato juice patch resulted in less demineralization compared to the hydrogen peroxide patch.

## Discussion

 Teeth comprise four distinct structures: enamel, dentin, and cementum, which are hard tissues, and pulp, a soft tissue.^14^ Enamel contains up to 96% calcium phosphate, water, and organic compounds. Dentin, meanwhile, contains approximately 70–75% calcium phosphate, 20% organic compounds, and 5%–10% water.^15,16^ Staining of teeth occurs in various forms based on its location and process. Extrinsic stains manifest on the tooth surface due to interactions between positively charged food particles and negatively charged tooth particles derived from salivary proteins. Factors contributing to color changes in teeth include tooth surface morphology, salivary protein composition, food and beverages consumed, smoking, and tooth hygiene levels. Several studies have linked the consumption of polyphenol-rich drinks such as tea, coffee, and red wine to extrinsic stains. Another type of staining, known as intrinsic discoloration, occurs when extrinsic stains penetrate the enamel and dentin, caused by substances such as food chromogens and tobacco products, which occurs due to the magnification of enamel porosity, allowing extrinsic stains to penetrate the enamel and dentin. Conversely, intrinsic stains occur when the discoloration is inside the tooth or penetrates beneath the enamel surface, which can result from various factors such as food molecules infiltrating damaged or perforated enamel, structural alterations in teeth or dental tissue thickness, the natural colors of enamel and dentin, systemic factors and metabolic diseases, trauma, and tetracycline exposure.^17,18^

 The cleansing methods for stained teeth are divided into two categories: physical and chemical. Physical cleansing involves the use of abrasive agents commonly formulated in toothpaste formulations. Effective abrasive agents are typically fine and spherical, as they can remove stains and impart a lustrous appearance to the teeth while minimizing dental caries caused by brushing. Commonly used abrasive agents include synthetic amorphous silica, calcium carbonate (shellfish derivatives), eggshell, calcite, natural lime, mica, dicalcium phosphate dihydrate, aluminum oxide, or bauxite.^17^ Moreover, products for tooth whitening have evolved into various categories, including whitening toothpastes, over-the-counter whitening strips and gels, whitening rinses, tray-based tooth whiteners, and in-office whitening. In most whitening dental products, the active ingredient is H_2_O_2_, which can be delivered as hydrogen peroxide or carbamide peroxide.^19^ H_2_O_2 _whitens teeth by oxidizing stains, which are organic compounds in enamel and dentin, rendering them colorless. It breaks down into free radicals, disrupting the double bond between the two carbon atoms (-C = C-) in the tooth stains.^17^

 In-office bleaching is a tooth-whitening method performed by professional dentists. While it effectively and rapidly whitens teeth, it can also cause undesired effects. The commonly used concentrations (30%–35% hydrogen peroxide) may cause a burning sensation in soft tissue (such as the mucosa), resulting in the discoloration of tissue to a whitish hue. To mitigate this, dentists often apply gum protectors to shield the patient’s tissues during the bleaching process. Similarly, at-home bleaching methods have been reported to cause irritation, primarily due to poorly fitting tooth molds rather than the bleaching agent itself. Despite lower concentrations of bleaching agents in at-home preparations, occasional gastrointestinal mucosal irritations are reported, including a burning sensation in the palate and throat and minor disturbances in the stomach or small intestine. Moreover, bleaching can affect tooth structure. Enamel may undergo pore enlargement, demineralization and decreased protein concentration, organic matrix degradation, changes in the calcium-phosphate ratio, and reduced calcium levels.^20^

 As tomatoes ripen and turn red, they accumulate hydrogen peroxide and peroxidase enzymes in their pericarp. This hydrogen peroxide can penetrate the layers of enamel and dentin, damaging organic molecules or stains, thereby achieving neutrality and oxidizing a wide range of colored organic and inorganic compounds. Consequently, this process results in the brightening of teeth. Furthermore, the peroxidase enzymes present in tomatoes can expedite this process by enhancing the speed of hydrogen peroxide in reducing color.^4,21^

 In addition, previous studies have demonstrated that tomato juice can impact tooth discoloration by soaking incisive teeth in tomato juice for varying durations.^22^ Meanwhile, a comparative study between tomato and apple juice highlighted the superior whitening effects of tomato juice.^5^ In this study, we found that the 65% tomato juice patch yielded the most favorable results compared to other concentrations. Several factors may influence tooth brightness, including enamel thickness, tooth age, and the pH of the whitening agent. Thicker enamel may prolong the penetration time of the whitening agent while aging can lead to thinner enamel layers and thicker dentin due to continuous secondary dentin formation. Additionally, lower pH levels can optimize the absorption of bleaching agents into the dentinal tubules due to tooth erosion.^23^

 Hydrogen peroxide is a potent oxidizing agent that is readily available. When hydrogen peroxide decomposes, it releases oxygen-free radicals that can react with both extrinsic and intrinsic stains, resulting in a bleaching effect.^24^ Despite being the most prevalent dental bleaching agent, it has drawbacks, such as reducing enamel hardness and causing irritation upon contact with soft tissues.^5^ Several studies have indicated that aggressive dental bleaching can exacerbate tooth sensitivity, alter the surface integrity and microstructure of enamel crystals, and increase susceptibility to demineralization.^18^

## Conclusion

 The tomato juice patch exhibited tooth bleaching potential with reduced erosive effects compared to the hydrogen peroxide patch. All concentrations of the tomato juice patch enhanced tooth brightness. Notably, the 65% tomato juice patch demonstrated the highest dental bleaching potential among all concentrations, with a lower demineralizing effect compared to the 12% hydrogen peroxide patch. Further research is needed to optimize its formulation and potential.

## Acknowledgments

 The authors thank Lisbeth Aswan, DDS from the Faculty of Dentistry, Moestopo University, who helped with the tooth specimens for this research.

## Competing Interests

 None.

## Ethical Approval

 This study was approved by the Ethics Committee, Faculty of Medicine UIN Syarif Hidayatullah Jakarta (No. B-026/F12/KEPK/TL.00/6/2023).
